# Improved Extraction Efficiency of Antioxidant Bioactive Compounds from *Tetraselmis chuii* and *Phaedoactylum tricornutum* Using Pulsed Electric Fields

**DOI:** 10.3390/molecules25173921

**Published:** 2020-08-27

**Authors:** Marialena Kokkali, Francisco J. Martí-Quijal, Mercedes Taroncher, María-José Ruiz, Katerina Kousoulaki, Francisco J. Barba

**Affiliations:** 1 Department of Nutrition and Feed Technology, Nofima AS, 5141 Bergen, Norway; marialena.kokkali@nofima.no; 2 Department of Preventive Medicine and Public Health, Food Science, Toxicology and Forensic Medicine, Faculty of Pharmacy, Universitat de València, Avda. Vicent Andrés Estellés, s/n 46100 Burjassot, València, Spain; francisco.j.marti@uv.es (F.J.M.-Q.); mercedes.taroncher@uv.es (M.T.); m.jose.ruiz@uv.es (M.-J.R.)

**Keywords:** pulsed electric fields, *Tetraselmis chuii*, *Phaeodactylum tricornutum*, antioxidant bioactive compounds, extraction

## Abstract

Pulsed electric fields (PEF) is a promising technology that allows the selective extraction of high-added value compounds by electroporation. Thus, PEF provides numerous opportunities for the energy efficient isolation of valuable microalgal bioactive substances (i.e., pigments and polyphenols). The efficiency of PEF-assisted extraction combined with aqueous or dimethyl sulfoxide (DMSO) solvents in recovering pigments and polyphenols from microalgae *Tetraselmis chuii* (*T. chuii*) and *Phaeodactylum tricornutum* (*P. tricornutum*) was evaluated. Two PEF treatments were applied: (1 kV/cm/400 pulses, 3 kV/cm/45 pulses), with a specific energy input of 100 kJ/kg. The total antioxidant capacity (TAC) was positively influenced by the use of DMSO. The highest TAC in the *T. chuii* culture was achieved at a lower extraction time and electric field than for *P. tricornutum*. The use of DMSO only improved the polyphenols′ extraction for *P. tricornutum,* whereas the PEF and extraction time were more important for *T. chuii*. Carotenoids and chlorophyll a were more efficiently extracted using DMSO, while chlorophyll b levels were higher following aqueous extraction for both microalgae. In *P. tricornutum,* the TAC and pigment extraction efficiency were in general higher at lower extraction times. It can be concluded that PEF may be a promising alternative for the enhancement of the selective extraction of antioxidant bioactive compounds from microalgae.

## 1. Introduction

Over the last years, several research studies have evaluated the potential of microalgae as a source of nutrients and bioactive compounds for food (e.g., *Spirulina*, *Chlorella*, *Nannochloropsis*, etc.) and feed applications. For example, microalgae have been used to obtain healthier food products by replacing protein of animal origin [1,2,3], as well as being a source of high added-value compounds in food additives and nutraceuticals [4,5,6].

One of the key steps in harnessing the potential of these microalgae is the extraction of the compounds, traditionally performed on dried biomass, using solvents [7]. However, the long extraction time, the co-extraction of undesirable compounds, as well as the increased downstream costs lead to the need for the development of more efficient and sustainable extraction technology alternatives [8,9,10].

In this sense, it has been shown that the use of pulsed electric fields (PEF), which is a technique consisting of the application of electrical pulses between two electrodes, has several advantages [6]. For instance, PEF lipid droplets remain intracellular after treatment, which provides advantages in selective processing [11] that targets the up-concentration of high value bioactive compounds. Moreover, the use of PEF being possible in aqueous solutions with a low dry matter content, opens opportunities for a more energy-efficient isolation of valuable microalgal compounds directly from the culture without the need for prior dewatering or drying [11].

Most of the studies evaluating the application of PEF focus on the use of aqueous suspensions of microalgae, thus mainly allowing extraction of water-soluble compounds [12,13], whereas the extraction of non-polar pigments (e.g., chlorophylls or carotenoids) is limited under these conditions. Some previous studies evaluated the use of supplementary extractions using 96% ethanol, obtaining considerable higher yields of pigments extracted from the PEF-pretreated microalgae *Chlorella vulgaris* [14]. More recently, the combination of PEF or ultrasound-pretreated microalgae suspensions and the binary mixture of water + organic solvent has been used to improve the extraction efficiency of pigments from microalgae *Nannochloropsis* spp. [15,16,17].

However, as reported in a previous review, the performance of extraction assisted by PEF varies greatly depending on the microalgae that is used [5]. This is why species-specific studies are necessary to generate the necessary information to be able to scale the process to an industrial level.

Most of the studies evaluating the PEF-assisted extraction from microalgae biomass have been focused on *Chlorella*, *Nannochloropsis*, etc., observing a lack of data on the PEF impact on the extraction of high added-value compounds from other microalgae species. *Tetraselmis chuii* (*T. chuii*) is a marine unicellular microalgae 12–14 μm in length and 9–10 μm in width belonging to the Chlamydomonadaceae family. The characteristics of the species are the ovoid shape of the cell and the four flagella that emerge from a depression near the apex. *Phaeodactylum tricornutum* (*P. tricornutum*) is a marine diatom 5–27 μm in length and 3–4 μm in width belonging to the Phaeodactylaceae family. *P. tricornutum* can be found in different morphotypes (i.e., fusiform, triradiate and oval) that may have different behaviors under different downstream processing conditions.

The novelty in the present work is mainly based on the use of a low electric field strength to promote microalgae electroporation, since most of the available works in the literature generally use high field strengths. Moreover, the PEF equipment used in this study allows for scaling up processing conditions to an industrial level. This also explains why the used field strength is low, since at an industrial level it is difficult to use high electric fields.

Therefore, the present research is aimed at the evaluation of the efficiency of PEF-assisted extraction combined with aqueous or dimethyl sulfoxide (DMSO) supplementary extraction in recovering high added-value compounds (pigments and total phenolic compounds) from *T. chuii* and *P. tricornutum*. 

## 2. Results and Discussion

**Figure 1:**
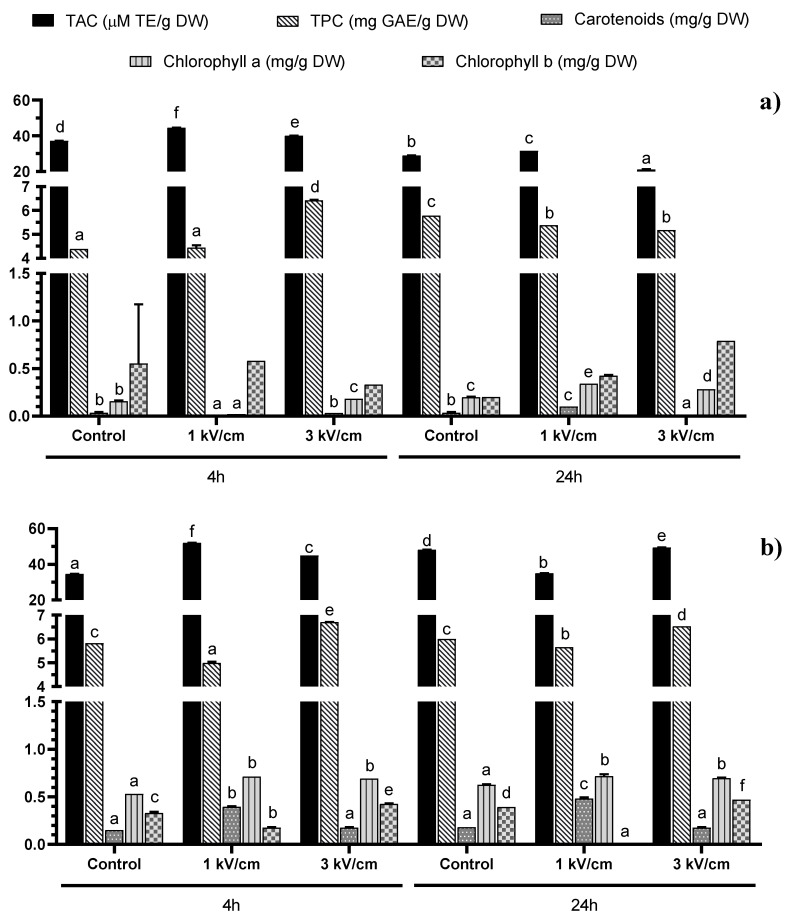
Total Antioxidant Capacity (TAC) and concentration of total phenolic compounds (TPC), total carotenoids, chlorophyll a and chlorophyll b in (**a**) water and (**b**) DMSO 50% in water extracts from *Tetraselmis chuii*. Bars with different letters in the same parameter indicate significant statistical differences (*p* < 0.05).

**Figure 2:**
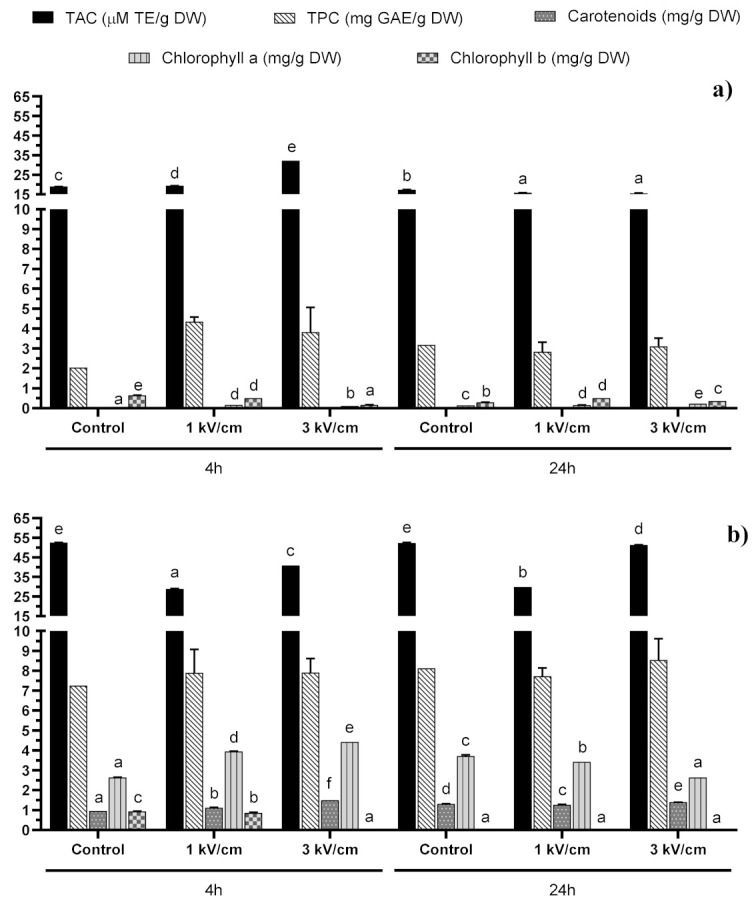
Total Antioxidant Capacity (TAC) and concentration of total phenolic compounds (TPC), total carotenoids, chlorophyll a and chlorophyll b in (**a**) water and (**b**) DMSO 50% in water extracts from *Phaeodactylum tricornutum*. Bars with different letters in the same parameter indicate significant statistical differences (*p* < 0.05).

In our study, the combined effects of the PEF treatment, extraction time and solvent type (polar: water vs. non-polar: DMSO) on antioxidant extraction from two microalgae species (i.e., *T. chuii* and *P. tricornutum*) were evaluated. It is known that polyphenols and pigments like carotenoids contribute significantly to the total antioxidant capacity (TAC) of microalgae [18]. For that reason, total phenolic compounds, carotenoids, chlorophyll a and chlorophyll b were also measured. The results on the TAC, total phenolics and pigments for *T. chuii* are summarized in Figure 1a for water and Figure 1b for DMSO, whereas the respective results for *P. tricornutum* are summarized in Figure 2a for water and Figure 2b for DMSO.

### 2.1. Total Antioxidant Capacity

The total antioxidant capacity (TAC) of the extracts measured by ABTS test also presented significant differences (*p* < 0.05). For instance, the analysis of variance (ANOVA) showed significant modifications in TAC according to the PEF treatment, solvent and time used for the extraction, independently of the microalgae evaluated (Figure 1 and Figure 2). For example, for *T. chuii*, the ABTS values were in the range of 21.13 to 51.97 μM TE/g dry weight (DW). The maximum value in *T. chuii* was reached after applying a 1 kV/cm treatment and supplementary extraction with DMSO during 4 h. On the other hand, for *P. tricornutum*, the TAC values varied between 15.58 and 51.36 μM TE/g DW, reaching the maximum value after applying 3 kV/cm and supplementary extraction in DMSO during 24 h. These values are in agreement with those obtained by other authors [18,19,20,21], who found TEAC values in the range of 13.36–48.90 μM TE/g DW and 6.79–67.93 μM TE/g DW when they evaluated the TAC of the extracts from *T. chuii* and *P. tricornutum*, respectively. It should be noted that, in general, TAC data may vary among different studies, not only according to the microalgae species studied but also the growth conditions used.

#### 2.1.1. Polyphenols

The total phenolic compounds (TPC) values in the *T. chuii* extracts ranged from 4.38 to 6.70 mg gallic acid equivalents (GAE)/g dry weight (DW), not observing any significant influence of the solvent used (Figure 1). Moreover, the maximum value was achieved with the PEF treatment at 3 kV/cm and 4 h of extraction, independently of the solvent used (water: 6.42 GAE/g DW and DMSO 50% in water: 6.70 mg GAE/g DW, respectively). 

However, in the case of *P. tricornutum*, we observed a significant influence of the solvent in relation to the recovery of antioxidant compounds (Figure 2). For instance, as shown in Figure 2, the values obtained after the aqueous extraction were significantly lower than those obtained after using DMSO. However, in this case, neither the field strength and pulses treatment nor the extraction time had any significant influence on the extraction of antioxidant compounds, since no significant differences were found. The maximum extraction yield was obtained after PEF pre-treatment + DMSO 50% in water, obtaining values ≈ 8 mg GAE/g DW. These values are in agreement with those obtained previously by Goiris et al. [18] for TPC after a conventional extraction with ethanol/water solvent; they extracted 3.74 mg GAE/g DW and 3.75 mg GAE/g DW for *Tetraselmis* spp. and *Phaeodactylum tricornutum*, respectively. Moreover, Safafar et al. [19] also found similar results when they evaluated the extraction of TPC from *P. tricornutum* using methanol as a solvent (3.16 mg GAE/g DW).

#### 2.1.2. Pigments

Finally, although the amount of total phenolic compounds obtained was similar, the amount of pigments was different between the two microalgae species studied, especially after the DMSO-assisted extraction (Figure 1 and Figure 2). This fact can be explained by the potential differences in the total pigment levels of both microalgae, as well as in the extraction efficiency resulting from their differences in cellular structure and size, the cells of *P. tricornutum* being slightly bigger than those of *T. chuii* and possessing different forms. *Tetraselmis chuii* cells have an oval form (10 × 14 μm), while *P. tricornutum* presents three different morphotypes [22]. These morphotypes are ovoid (10–12 μm in length), triradiate (15–20 μm) and fusiform (20–30 μm in length, with a diameter of 1–3 μm) [23]. The difference in the size and form of the cells may be the reason behind the higher pigment extraction efficiency in *P. tricornutum,* demonstrating that both the shape and size of the cell may influence the effect of electroporation by PEF. Moreover, for elongated cells like the fusiform morphotypes of *P. tricornutum*, their orientation in the medium with respect to the electric fields may also influence the electroporation efficiency, depending on which side (the long or the short one) is positioned parallel to the electrodes [24]. Nevertheless, in the case of *T. chuii*, as the cells have a more spherical form, their orientation may be less important.

The composition of the cell wall must also be considered, since it is an important factor that can influence extraction [25]. Contrary to what is usual in diatoms, the *P. tricornutum* cell wall is mainly composed of sulphated glucuronomannan and is poor in silica, especially in its triradiate and fusiform morphotypes, which makes them softer [23,26]. On the other hand, the *T. chuii* cell wall is rich in a pectin-like polysaccharide, possibly conferring a relatively higher resistance to the cells [27].

The total carotenoids and chlorophyll a were more efficiently extracted using DMSO than using water for both microalgae species studied, while the analyzed chlorophyll b levels were higher following the aqueous extraction. The extraction time did not significantly influence the recovery of these compounds. In *T. chuii*, the highest recovery of carotenoids (0.48 mg/g DW) was observed after 24 h extraction using DMSO as the solvent and a PEF at 1 kV/cm and 400 pulses.

Regarding chlorophyll a, all the samples treated with PEF presented a higher extraction than the control samples. Regarding the extraction of chlorophyll b, the optimal treatment for the highest recovered amount (0.79 mg/g DW) was found after applying PEF (3 kV/cm, 45 pulses) followed by 24 h of extraction using water. In this case, no significant differences were found, but this was probably due to the high variability of one of the treatments (Control 4 h).

In *P. tricornutum*, the highest carotenoid yield (1.5 mg/g DW) was observed after a PEF pre-treatment (3 kV/cm, 45 pulses) followed by 4 h of extraction in DMSO 50% in water. The highest concentration of chlorophyll a (4.42 mg/g DW) was found after 4 h and a PEF pre-treatment (3 kV/cm, 45 pulses) using DMSO 50% in water as the solvent media. Finally, the highest recovery of chlorophyll b was obtained in control samples after 4 h of extraction, while the PEF treatment decreased the yield of the process, especially at 3 kV/cm. These results are in agreement with Parniakov et al. [15], who obtained values in the same range to those found in our study when they evaluated the total carotenoids and chlorophylls extraction after a PEF pre-treatment (20 kV/cm, 400 pulses of 10 μs) and a subsequent extraction using binary mixtures of DMSO or ethanol at 50% in a water solution for *Nannochloropsis* spp. microalgae. 

Moreover, in another study, Parniakov et al. [17] also measured the values for carotenoids and chlorophylls, again obtaining similar values for *Nannochloropsis* spp. after a PEF pre-treatment (20 kV/cm, 160 J per pulse and a treatment duration between 0.01 and 6 ms). Luengo et al. [14] obtained an increase of 124, 164 and 218% in the extraction of carotenoids, chlorophyll a and chlorophyll b, respectively, from *Chlorella vulgaris* after applying a PEF treatment (20 kV/cm for 75 μs). However, the same authors did not find any significant improvement in the extraction of these pigments when they applied 15 kV/cm or lower electric fields. In addition, the values for total carotenoids, chlorophyll a and chlorophyll b obtained by these authors after a PEF pre-treatment and subsequent ethanolic extraction were in the same range as our results for aqueous extracts (0–2 mg/g DW). In contrast, Pataro et al. [28] obtained higher values for carotenes (41.8 mg/g DW) and chlorophyll a (60.2 mg/g DW) when they treated *Nannochloropsis oceanica* at 10 kV/cm and 100 kJ/kg. Finally, Safafar et al. [19] obtained higher values for total carotenoids (2.92–6.70 mg/g) in methanolic extracts for Chlorophyta microalgae (*Chlorella* sp., *Dunaniella* and *Desmodesmus*) and 4.60 mg/g for *P. tricornutum*. However, the values for chlorophyll a and b were closer to our results. Chlorophyll a reached values from 0.62 to 3.42 mg/g for *Chlorella* sp., *Dunaniella* and *Desmodesmus*, and 2.71 mg/g for *P. tricornutum*. On the other hand, they extracted from 0.39 to 1.45 mg/g of chlorophyll b from Chlorophyta microalgae, while they did not detect any chlorophyll b in *P. tricornutum* methanolic extracts.

In our study, the yield of pigment extraction was low in comparison to other studies. This may be due to the high water concentrations we used. Leonhardt et al. [29] had a reduction of 50% in the yield of chlorophyll extraction when they used EtOH 70% in water instead of pure ethanol. Moreover, the extraction of chlorophylls also decreased by 67% when they used DMSO 70% in water rather than pure DMSO. Pigments can be found within the cell, both outside and inside organelles such as chloroplasts [30]. Thus, another relevant factor that may have influenced the pigment extraction efficiency was the field strength used in the PEF pre-treatment. Pore formation is directly related to the intensity of the treatment. More specifically, the electric field is inversely related to the size of the cell membrane. Therefore, to create pores in intracellular organelles such as chloroplasts, a higher electric field is necessary. In this sense, it has been seen that the electric field required to electroporate organelles is 100 kV/cm [6]. On the other hand, it has been seen that, in order to achieve electroporation in the cells of some microalgae such as *Chlorella vulgaris*, a treatment greater than 10–15 kV/cm with pulses lasting in the microseconds range was adequate [6,14]. Moreover, it has been found that pore formation can also be promoted using a lower electric field strength by increasing the duration of the electric pulse to the millisecond range [31]. Therefore, this may explain our results, where we observed a lower pigment concentration in the extracts obtained, probably due to the fact that part of the pigments present in the intracellular medium were extracted, while those present in the organelles were not, due to the low-intensity electric field strength applied.

In our results, there is a clear increase in the pigment extraction yield when DMSO was used as a solvent. This is in agreement with previous studies [15,19,32] and is explained by the hydrophobic hydrocarbon structure of both the carotenoids and chlorophylls studied, which renders them mainly soluble in non-polar solvents.

## 3. Materials and Methods 

### 3.1. Chemicals and Reagents

The ABTS (2,2′-Azino-Bis-3-Ethylbenzothiazoline-6-Sulfonic Acid), Folin–Ciocalteu reagent, gallic acid, Trolox (6-hydroxy-2,5,7,8-tetramethylchroman-2-carboxylic acid) and potassium persulfate (K_2_S_2_O_8_) were purchased from Sigma–Aldrich (Steinheim, Baden-Württemberg, Germany). Sodium carbonate (Na_2_CO_3_) and dimethyl sulfoxide (DMSO) were acquired from VWR (Saint-Prix, France). Ethanol (99.9%) and methanol (95%) were obtained from Baker (Deventer, Overijssel, The Netherlands).

### 3.2. Samples

*T. chuii* and *P. tricornutum* were produced in four 800 L GemTube (LGEM, Rotterdam, The Netherlands) photobioreactors at the National Algae pilot plant in Mongstad (NAM), Norway. The photobioreactors were located in a greenhouse exposed to natural light and additionally equipped with artificial illumination (EAX 170W LED lights, Evolys AS, Oslo, Norway) with an average incident artificial light of 200 μmol m^−2^·s^−1^. The *P. tricornutum* biomass used in this work’s studies was produced in May–June 2017, whereas the *T. chuii* biomass was produced in July–October 2017. The reactors were operated at pH 7.8 by on-demand CO_2_ addition, and culture temperatures were maintained between 15 and 35 °C by heating the greenhouse or spraying the reactors with water to cool them down. The reactors were operated in dual mode, so that mixing was provided by both a liquid pump and air pump, resulting in a liquid velocity of approximately 0.3 m·s^−1^. The microalgae were cultivated in modified WUR medium, which was based on natural seawater (Fensfjorden, Mongstad, salinity of 31 ppt) enriched with a nutrient stock solution. The seawater was chemically sterilized (sodium hypochlorite), and active chlorite was deactivated by filtration through active carbon, which was followed by filtration (1 μm).

The microalgae biomass was produced in a fed-batch process: the reactors were harvested once per week (between 50–90% of the culture volume), after which seawater and nutrients were added to compensate for the volume taken. After harvesting, the biomass was dewatered using a spiral plate centrifuge (Evodos 25, Evodos b.v., Raamsdonksveer, The Netherlands), resulting in a paste of approx. 22% dry weight in the case of *P. tricornutum* and approx. 35% dry weight in the case of *T. chuii*. The paste was vacuum-packed and directly frozen at −20 °C before sending it to Nofima in Bergen, Norway and storing it at −20 °C until further use.

### 3.3. Pulsed Electric Fields (PEF)-Assisted Extraction

For the PEF treatment of the biomass (freeze-dried biomass of *T. chuii* and frozen paste of *P. tricornutum*), the PEF-Cellcrack III (German Institute of Food Technologies (DIL)) equipment (ELEA, Quakenbrück, Germany) was used. For the preparation of each sample, 198 g of tap water were added to 2 g of biomass to end up with a microalgae biomass solution of 1% DM, according to Parniakov et al. [15]. A chamber of 900 mL capacity was chosen, the gap between the electrodes was set at 10 cm, and the mass added in the cell was always 200 g. The specific energy input was 100 kJ/kg; the number of pulses was 45 or 400 pulses, depending on the voltage applied (3 or 1 kV/cm, respectively). Before and after treatment, the temperature and conductivity were measured in the sample with a portable conductivity meter ProfiLine Cond 3310 (WTW, Xylem Analytics, Weilheim in Oberbayern, Germany). 

In both PEF pre-treatments that we applied, the specific energy input, pulse duration and frequency remained constant at 100 kJ/kg, 100 ms and 2 Hz, whereas the field strength and the number of pulses varied in the different treatments. The minimum electric field strength necessary to produce changes in cells is 1 kV/cm, and it has been found that with a pulse duration of milliseconds an electric field of 3–4 kV/cm is enough to create electroporation [31,33]. Based on these data, we decided to compare a low electric field (1 kV/cm) with a moderate one (3 kV/cm). Therefore, the parameters of the first treatment consisted of 45 pulses with a field strength of 3 kV/cm, and the energy per pulse delivered to the treated suspension was 450 J. The second one consisted in 400 pulses at 1 kV/cm, and in this case the energy per pulse delivered to the treated suspension was just 50 J. Thus, a treatment of moderate intensity and short duration and another one of low intensity and long duration were compared in our study. Moreover, we decided to use 1 and 3 kV/cm because 3 kV/cm is the maximum electric field that can be applied by the PEF equipment used in this study. As discussed before, high field strengths are difficult to use in the industry. The PEF conditions chosen in our study had a relatively low specific energy (100 kJ/kg). The specific energy input influences the degree of membrane permeabilization [34,35]. Some authors have seen how specific energy values greater than 50–100 kJ/kg only slightly increased the intracellular content release when they applied treatments at field strengths of 27 or 35 kV/cm [36]. Moreover, in a study by Töpfl, a specific energy of 100 kJ/kg and a field strength of 15 kV/cm were enough to increase the extraction of carotenoids from the microalgae *Chlorella vulgaris* and *Spirulina platensis* [37]. 

The strategy based on the design of experiments has not been used because the objective of the present study was not to optimize the extraction of compounds. The main objective of this work was to compare the effect of a very low (1 kV/cm) electric field with that of another moderate/low (3 kV/cm) one. For this reason, experiments modifying the electric field strength were selected. The control sample (without pre-treatment with PEF) can be established as a solid-liquid extraction, a conventional extraction technique widely used in the industry. Therefore, comparing the results with the control means comparing the use of PEF technology with conventional extraction.

### 3.4. Supplementary Solvent Extraction

After the PEF treatment, solvents (Dimethyl Sulfoxide (DMSO) or distilled water (dH_2_O)) were added into the samples at 1:1 *v*/*v* with the scope to further enhance nutrient extraction. After the solvents were added, the samples were stirred with rotating magnets at 400 rpm for either 4 or 24 h at room temperature to test the effect of the stirring time on the compound extractability from the processed biomasses. After mixing, the samples were centrifuged for 10 min at 4000 rpm using a 5810R centrifuge (Eppendorf AG). The supernatant was collected and kept frozen at −20 °C for further analysis. Each sample was processed in each setting in duplicate.

### 3.5. Chemical Analyses

#### 3.5.1. Total Phenolic Compounds (TPC) 

For the determination of TPC (mg of gallic acid equivalent (GAE)/g DW), the Folin−Ciocalteu method, previously described by Parniakov et al. [17], was used. This technique is based on the colorimetric oxidation/reduction reaction of phenols [38] using gallic acid (Sigma−Aldrich, Steinheim, Germany) as standard. First, 50% *v*/*v* Folin-Ciocalteu reagent, 2% Na_2_CO_3_, as well as the diluted gallic acid standards were prepared. Then, 100 μL of sample extract was mixed with 3 mL of Na_2_CO_3_, and finally 100 μL of Folin-Ciocalteu reagent were added to this mixture. The samples were incubated for 1 h at room temperature. The absorbance was measured at a wavelength of 750 nm using a spectrophotometer Perkin-Elmer UV/Vis Lambda 2 spectrophotometer (Perkin-Elmer, Rodgau-Jügesheim, Germany). All analyses were performed in triplicate. If differences between parallels exceeded 5%, new duplicate analyses were carried out.

#### 3.5.2. Trolox Equivalent Antioxidant Capacity (TEAC)

In order to determine the total antioxidant capacity (TAC), the Trolox Equivalent Antioxidant Capacity (TEAC) assay was used. The value of TEAC (millimolar Trolox equivalent, mM TE) measures the antioxidant capacity of a given substance, as compared to the standard, Trolox (Sigma-Aldrich, Steinheim, Germany). The TEAC was measured using the method previously reported by Re et al. [39], based on the use of the ABTS radical decolorization assay.

The ABTS radical cations (ABTS^+^) are produced by reacting ABTS 7 mM of a stock solution with 140 mM potassium persulfate (K_2_S_2_O_8_). The mixture was kept in darkness at room temperature for 12–16 h before use. The solution was then diluted with ethanol until an absorbance of 0.70 ± 0.02 was reached at 734 nm. Once the necessary absorbance was reached, 2 mL of ABTS·+ were mixed with 100 μL of extract, and the sample was incubated for 20 min at 20 °C. The absorbance was measured at a wavelength of 734 nm in a Perkin-Elmer UV/Vis Lambda 2 spectrophotometer (Perkin-Elmer, Rodgau-Jügesheim, Germany). All analyses were performed in triplicate. If differences between parallels exceeded 5%, new duplicate analyses were carried out.

### 3.6. Total Carotenoids, Chlorophyll a and b

The carotenoid and chlorophyll contents were estimated spectrophotometrically according to the study of Parniakov et al. [16], which was based on the method previously reported by Arnon and by Lichtethaler and Wellburn [40,41]. This method is based on the determination of the carotenoid and chlorophyll contents based on the maximum absorbances of chlorophyll a ($A_{ch}^{a}$), chlorophyll b ($A_{ch}^{b}$) and total carotenoids ($A_{cr}$), which are found at the wavelengths of *λ* ≈ 665 nm, ≈ 653 nm and ≈ 470 nm, respectively [42]. Aliquots of the extracts were diluted 15–300 times with 90% (*v*/*v*) methanol in water, and the absorbance (A) was measured at the 470, 653 and 665 nm wavelengths. The carotenoid, chlorophyll a and chlorophyll b contents were calculated according to the equations of Arnon, Lichtethaler and Wellburn [40,41] as follows:

(1) $$C_{ch}^{a}\;=\;16.82A_{ch}^{a}\;-\;9.28A_{ch}^{b}$$

(2) $$C_{ch}^{b}=36.92A_{ch}^{b}\;-16.54A_{ch}^{a}$$

(3) $$C_{cr}=(1000A_{cr}-1.91C_{ch}^{a}-95.15C_{ch}^{b})/225$$

where $C_{ch}^{a}$, $C_{ch}^{b}$ and $C_{cr}$ are the concentrations (mg of pigment/g DW) of chlorophyll a, chlorophyll b, and total carotenoids, respectively. 

### 3.7. Statistical Analyses

The data were analyzed using an analysis of variance (ANOVA), where the PEF pre-treatment, use of solvent and time of extraction were set as factors and the compound concentrations were set as variables. Data were expressed as mean ± standard deviation (SD) values. A probability value of *p* < 0.05 was considered significant. Furthermore, an LSD test was used to determine differences between treatment means. All statistical analyses were performed using the software GraphPad Prism 8.0.2 (GraphPad Software, San Diego, CA, USA).

## 4. Conclusions

This study highlights the potential of PEF pre-treatment to improve antioxidant compound extraction from untreated microalgae biomass. The PEF treatment, solvent type (polar vs. non-polar) and extraction time significantly affected the extraction of carotenoids and phenolic compounds, as well as the TAC, from *T. chuii* and *P. tricornutum* biomasses. Our study demonstrated that PEF may be a promising alternative for enhancing the selective extraction of antioxidant bioactive compounds from microalgae, which could be interesting for industrial upscaling. *P. tricornutum* showed the best pigment extraction, with a PEF pre-treatment and a subsequent extraction with a binary mixture of DMSO 50% in water. Both microalgae extracts possessed a similar and relatively high antioxidant capacity, especially after the non-polar extraction.

## References

[1] Thirumdas R., Brnćić M., Brnćić S.R., Barba F.J., Gálvez F., Zamuz S., Lacomba R., Lorenzo J.M. (2018). Evaluating the impact of vegetal and microalgae protein sources on proximate composition, amino acid profile, and physicochemical properties of fermented Spanish “chorizo” sausages. J. Food Process. Preserv..

[2] Marti-Quijal F.J., Zamuz S., Tomašević I., Gómez B., Rocchetti G., Lucini L., Remize F., Barba F.J., Lorenzo J.M. (2019). Influence of different sources of vegetable, whey and microalgae proteins on the physicochemical properties and amino acid profile of fresh pork sausages. LWT.

[3] Marti-Quijal F.J., Zamuz S., Tomašević I., Rocchetti G., Lucini L., Marszałek K., Barba F.J., Lorenzo J.M. (2019). A chemometric approach to evaluate the impact of pulses, Chlorella and Spirulina on proximate composition, amino acid, and physicochemical properties of turkey burgers. J. Sci. Food Agric..

[4] Barba F.J. (2017). Microalgae and seaweeds for food applications: Challenges and perspectives. Food Res. Int..

[5] Geada P., Rodrigues R., Loureiro L., Pereira R., Fernandes B., Teixeira J.A., Vasconcelos V., Vicente A.A. (2018). Electrotechnologies applied to microalgal biotechnology—Applications, techniques and future trends. Renew. Sustain. Energy Rev..

[6] Poojary M.M., Barba F.J., Aliakbarian B., Donsì F., Pataro G., Dias D.A., Juliano P. (2016). Innovative alternative technologies to extract carotenoids from microalgae and seaweeds. Mar. Drugs.

[7] Ambrozova J.V., Misurcova L., Vicha R., Machu L., Samek D., Baron M., Mlcek J., Sochor J., Jurikova T. (2014). Influence of extractive solvents on lipid and fatty acids content of edible freshwater algal and seaweed products, the green microalga *Chlorella kessleri* and the cyanobacterium *Spirulina platensis*. Molecules.

[8] Carullo D., Abera B.D., Casazza A.A., Donsì F., Perego P., Ferrari G., Pataro G. (2018). Effect of pulsed electric fields and high pressure homogenization on the aqueous extraction of intracellular compounds from the microalgae *Chlorella vulgaris*. Algal Res..

[9] Günerken E., D’Hondt E., Eppink M.H.M., Garcia-Gonzalez L., Elst K., Wijffels R.H. (2015). Cell disruption for microalgae biorefineries. Biotechnol. Adv..

[10] Luengo E., Martínez J.M., Bordetas A., Álvarez I., Raso J. (2015). Influence of the treatment medium temperature on lutein extraction assisted by pulsed electric fields from *Chlorella vulgaris*. Innov. Food Sci. Emerg. Technol..

[11] Eing C., Goettel M., Straessner R., Gusbeth C., Frey W. (2013). Pulsed electric field treatment of microalgae—Benefits for microalgae biomass processing. IEEE Trans. Plasma Sci..

[12] Grimi N., Dubois A., Marchal L., Jubeau S., Lebovka N.I., Vorobiev E. (2014). Selective extraction from microalgae *Nannochloropsis* sp. using different methods of cell disruption. Bioresour. Technol..

[13] Martínez J.M., Delso C., Álvarez I., Raso J. (2020). Pulsed electric field-assisted extraction of valuable compounds from microorganisms. Compr. Rev. Food Sci. Food Saf..

[14] Luengo E., Condon-Abanto S., Alvarez I., Raso J. (2014). Effect of pulsed electric field treatments on permeabilization and extraction of pigments from *Chlorella vulgaris*. J. Membr. Biol..

[15] Parniakov O., Barba F.J., Grimi N., Marchal L., Jubeau S., Lebovka N., Vorobiev E. (2015). Pulsed electric field assisted extraction of nutritionally valuable compounds from microalgae *Nannochloropsis* spp. using the binary mixture of organic solvents and water. Innov. Food Sci. Emerg. Technol..

[16] Parniakov O., Apicella E., Koubaa M., Barba F.J., Grimi N., Lebovka N., Pataro G., Ferrari G., Vorobiev E. (2015). Ultrasound-assisted green solvent extraction of high-added value compounds from microalgae *Nannochloropsis* spp.. Bioresour. Technol..

[17] Parniakov O., Barba F.J., Grimi N., Marchal L., Jubeau S., Lebovka N., Vorobiev E. (2015). Pulsed electric field and pH assisted selective extraction of intracellular components from microalgae *Nannochloropsis*. Algal Res..

[18] Goiris K., Muylaert K., Fraeye I., Foubert I., De Brabanter J., De Cooman L. (2012). Antioxidant potential of microalgae in relation to their phenolic and carotenoid content. J. Appl. Phycol..

[19] Safafar H., van Wagenen J., Møller P., Jacobsen C. (2015). Carotenoids, phenolic compounds and tocopherols contribute to the antioxidative properties of some microalgae species grown on industrial wastewater. Mar. Drugs.

[20] German-Báez L.J., Valdez-Flores M.A., Félix-Medina J.V., Norzagaray-Valenzuela C.D., Santos-Ballardo D.U., Reyes-Moreno C., Shelton L.M., Valdez-Ortiz A. (2017). Chemical composition and physicochemical properties of *Phaeodactylum tricornutum* microalgal residual biomass. Food Sci. Technol. Int..

[21] Norzagaray-Valenzuela C.D., Valdez-Ortiz A., Shelton L.M., Jiménez-Edeza M., Rivera-López J., Valdez-Flores M.A., Germán-Báez L.J. (2017). Residual biomasses and protein hydrolysates of three green microalgae species exhibit antioxidant and anti-aging activity. J. Appl. Phycol..

[22] Cagney N., Zhang T., Bransgrove R., Allen M.J., Balabani S. (2017). Effects of cell motility and morphology on the rheology of algae suspensions. J. Appl. Phycol..

[23] Francius G., Tesson B., Dague E., Martin-Jézéquel V., Dufrêne Y.F. (2008). Nanostructure and nanomechanics of live *Phaeodactylum tricornutum* morphotypes. Environ. Microbiol..

[24] Lebovka N., Vorobiev E., Miklavcic D. (2017). Mathematical models of pulsed electric field treatment of plant tissues and simulation of related phenomena. Handbook of Electroporation.

[25] Barba F.J., Grimi N., Vorobiev E. (2014). New approaches for the use of non-conventional cell disruption technologies to extract potential food additives and nutraceuticals from microalgae. Food Eng. Rev..

[26] Le Costaouëc T., Unamunzaga C., Mantecon L., Helbert W. (2017). New structural insights into the cell-wall polysaccharide of the diatom *Phaeodactylum tricornutum*. Algal Res..

[27] Suarez Garcia E., Lo C., Eppink M.H.M., Wijffels R.H., van den Berg C. (2019). Understanding mild cell disintegration of microalgae in bead mills for the release of biomolecules. Chem. Eng. Sci..

[28] Pataro G., Carullo D., Ferrari G. (2019). PEF-assisted supercritical CO_2_ extraction of pigments from microalgae nannochloropsis oceanica in a continuous flow system. Chem. Eng. Trans..

[29] Leonhardt L., Käferböck A., Smetana S., de Vos R., Toepfl S., Parniakov O. (2020). Bio-refinery of *Chlorella sorokiniana* with pulsed electric field pre-treatment. Bioresour. Technol..

[30] Zhang R., Lebovka N., Marchal L., Vorobiev E., Grimi N. (2020). Pulsed electric energy and ultrasonication assisted green solvent extraction of bio-molecules from different microalgal species. Innov. Food Sci. Emerg. Technol..

[31] Luengo E., Raso J., Miklavcic D. (2017). Pulsed electric field-assisted extraction of pigments from chlorella vulgaris. Handbook of Electroporation.

[32] Zhang R., Parniakov O., Grimi N., Lebovka N., Marchal L., Vorobiev E. (2019). Emerging techniques for cell disruption and extraction of valuable bio-molecules of microalgae *Nannochloropsis* sp.. Bioprocess. Biosyst. Eng..

[33] Martínez J.M., Gojkovic Z., Ferro L., Maza M., Álvarez I., Raso J., Funk C. (2019). Use of pulsed electric field permeabilization to extract astaxanthin from the Nordic microalga *Haematococcus pluvialis*. Bioresour. Technol..

[34] Frey W., Gusbeth C., Schwartz T. (2013). Inactivation of *Pseudomonas putida* by pulsed electric field treatment: A study on the correlation of treatment parameters and inactivation efficiency in the short-pulse range. J. Membr. Biol..

[35] Goettel M., Eing C., Gusbeth C., Straessner R., Frey W. (2013). Pulsed electric field assisted extraction of intracellular valuables from microalgae. Algal Res..

[36] Pataro G., Goettel M., Straessner R., Gusbeth C., Ferrari G., Frey W. (2017). Effect of PEF treatment on extraction of valuable compounds from microalgae *C. vulgaris*. Chem. Eng. Trans..

[37] Töpfl S. (2006). Pulsed Electric Fields (PEF) for Permeabilization of Cell Membranes in Food-and Bioprocessing-Applications, Process and Equipment Design and Cost Analysis. Ph.D. Thesis.

[38] Singleton V.L., Orthofer R., Lamuela-Raventos R.M. (1999). Analysis of total phenols and other oxidation substrates and antioxidants by means of Folin-Ciocalteau reagent. Methods Enzymol..

[39] Re R., Pellegrini N., Proteggente A., Pannala A., Yang M., Rice-Evans C. (1999). Antioxidant activity applying an improved ABTS radical cation decolorization assay. Free Radic. Biol. Med..

[40] Lichtethaler H.K., Wellburn A.R. (1983). Determinations of total carotenoids and chlorophylls a and b of leaf extracts in different solvents. Biochem. Soc. Trans..

[41] Arnon D.I. (1949). Copper enzymes in isolated chloroplasts, polyphenoxidase in *Beta vulgaris*. Plant. Physiol..

[42] Kumar P., Ramakritinan C.M., Kumaraguru A.K. (2010). Solvent extraction and spectrophotometric determination of pigments of some algal species from the shore of puthumadam, southeast coast of India. Int. J. Ocean. Oceanogr..

